# A Comparative Phase I Study of Combination, Homologous Subtype-C DNA, MVA, and Env gp140 Protein/Adjuvant HIV Vaccines in Two Immunization Regimes

**DOI:** 10.3389/fimmu.2017.00149

**Published:** 2017-02-22

**Authors:** Sarah Joseph, Killian Quinn, Aldona Greenwood, Alethea V. Cope, Paul F. McKay, Peter J. Hayes, Jakub T. Kopycinski, Jill Gilmour, Aleisha N. Miller, Christof Geldmacher, Yuka Nadai, Mohamed I. M. Ahmed, David C. Montefiori, Len Dally, George Bouliotis, David J. M. Lewis, Roger Tatoud, Ralf Wagner, Mariano Esteban, Robin J. Shattock, Sheena McCormack, Jonathan Weber

**Affiliations:** ^1^MRC Clinical Trials Unit at UCL, Institute of Clinical Trials and Methodology, University College London, London, UK; ^2^Department of Medicine, Imperial College London, London, UK; ^3^Clinical Research Centre, University of Surrey, Guildford, UK; ^4^IAVI Human Immunology Laboratory, Imperial College London, London, UK; ^5^ICTU, Department of Public Health, Imperial College London, London, UK; ^6^Department of Infectious Diseases and Tropical Medicine, Klinikum of the University of Munich, Munich, Germany; ^7^German Centre for Infection Research (DZIF), Munich, Germany; ^8^Duke University Medical Centre, Durham, NC, USA; ^9^The EMMES Corporation, Rockville, MD, USA; ^10^Clinical Research Facility, Imperial College Healthcare NHS Trust, London, UK; ^11^University of Regensburg and University Hospital Regensburg, Regensburg, Germany; ^12^National Center for Biotechnology, CSIC, Madrid, Spain

**Keywords:** HIV vaccine, phase I trial, DNA, MVA, envelope protein

## Abstract

There remains an urgent need for a prophylactic HIV vaccine. We compared combined MVA and adjuvanted gp140 to sequential MVA/gp140 after DNA priming. We expected Env-specific CD4+ T-cells after DNA and MVA priming, and Env-binding antibodies in 100% individuals after boosting with gp140 and that combined vaccines would not compromise safety and might augment immunogenicity. Forty volunteers were primed three times with DNA plasmids encoding (CN54) env and (ZM96) gag-pol-nef at 0, 4 and 8 weeks then boosted with MVA-C (CN54 env and gag-pol-nef) and glucopyranosyl lipid adjuvant—aqueous formulation (GLA-AF) adjuvanted CN54gp140. They were randomised to receive them in combination at the same visit at 16 and 20 weeks (accelerated) or sequentially with MVA-C at 16, 20, and GLA-AF/gp140 at 24 and 28 weeks (standard). All vaccinations were intramuscular. Primary outcomes included ≥grade 3 safety events and the titer of CN54gp140-specific binding IgG. Other outcomes included neutralization, binding antibody specificity and T-cell responses. Two participants experienced asymptomatic ≥grade 3 transaminitis leading to discontinuation of vaccinations, and three had grade 3 solicited local or systemic reactions. A total of 100% made anti-CN54gp140 IgG and combining vaccines did not significantly alter the response; geometric mean titer 6424 (accelerated) and 6578 (standard); neutralization of MW965.2 Tier 1 pseudovirus was superior in the standard group (82 versus 45% responders, *p* = 0.04). T-cell ELISpot responses were CD4+ and Env-dominant; 85 and 82% responding in the accelerated and standard groups, respectively. Vaccine-induced IgG responses targeted multiple regions within gp120 with the V3 region most immunodominant and no differences between groups detected. Combining MVA and gp140 vaccines did not result in increased adverse events and did not significantly impact upon the titer of Env-specific binding antibodies, which were seen in 100% individuals. The approach did however affect other immune responses; neutralizing antibody responses, seen only to Tier 1 pseudoviruses, were poorer when the vaccines were combined and while T-cell responses were seen in >80% individuals in both groups and similarly CD4 and Env dominant, their breadth/polyfunctionality tended to be lower when the vaccines were combined, suggesting attenuation of immunogenicity and cautioning against this accelerated regimen.

## Introduction

In an era of antiretroviral medication for the treatment and prevention of HIV, concerns around access, toxicity, and escalating cost suggest that a vaccine for HIV is still likely to be the most effective and sustainable way of reducing new infections (1, 2). Of the five HIV efficacy vaccine trials to date, there has only been only one to demonstrate significant, if modest efficacy; the RV144 “Thai” trial (3–8). This study with 16,402 subjects randomized to four immunizations with ALVAC given twice and then twice more with AIDSVAX B/E adjuvanted with ALUM, reported 31.2% protection (95% CI 1–51) against acquisition, without impacting HIV viral load or CD4+ T cell count (8). Subsequent immunological analyzes reported an inverse correlation between the levels of circulating polyclonal non-neutralizing antibodies and risk of infection, which has been associated with Fc receptor-mediated antibody effector functions (9–15). The results stimulated interest in prime-boost pox and protein combination vaccine approaches and the role of non-neutralizing antibodies.

Heterologous prime boost regimens employing DNA, viral vectors, and/or recombinant proteins have generated robust cellular and humoral responses maximizing breadth and potency while limiting the attenuating effects of vector specific immunity (16–20). DNA vaccines have been shown to prime cellular and humoral immune responses, upon boosting with recombinant vectors (21). The EuroVacc trials demonstrated that DNA prime, NYVAC boost increased the frequency, magnitude, and breadth of HIV-specific T-cell ELISpot responses (22, 23) and that three DNA priming immunizations were more immunogenic than two (24). A recent clinical trial comparing different prime boost regimens showed no benefit of DNA priming for Env-specific antibody responses but evidence of an improvement in T-cell responses, although overall immunogenicity was lower than seen previously in response to the same DNA and MVA vaccines (25).

In this study, the UK HIV Vaccine Consortium built upon these prior data showing enhanced immunogenicity of DNA prime, pox vector boost, and the protection seen in RV144 by protein boosting, to produce homologous DNA, MVA, and gp140 immunogens. We have made DNA plasmids and an MVA expressing matched HIV-1 subtype C (CN54)-derived inserts, and adjuvanted trimeric glycoprotein with a view for use in Sub-Saharan Africa. We believe this strategy is ideally suited to inducing Env-dominant CD4+ T-cell responses, favoring the development of high titer Env-specific antibody responses. The same trimeric recombinant CN54gp140 protein has already been administered to 469 individuals in a variety of trial settings (with and without DNA priming, or adjuvant and via different routes), showing excellent safety and induction of vaccine specific antibodies (26–28). When given systemically with glucopyranosyl lipid adjuvant—aqueous formulation (GLA-AF) after priming with heterologous DNA and MVA, high titer systemic binding antibodies were seen to the protein (28).

Prompted by the results of the RV144 trial, but with long-term feasibility in mind, we have explicitly assessed the impact of combining pox (MVA-C) and GLA-AF adjuvanted CN54gp140 protein after priming with DNA. We compared the safety and immunogenicity of two regimens using identical vaccines; given sequentially in one regimen (standard) and with the pox and protein combined in the other (accelerated). We shortened the regimens relative to our previous studies and RV144 by reducing the intervals between vaccinations, with 4 weeks between each of three DNA immunizations, 8 weeks between prime and first boost, and 4 weeks between subsequent boosts. We administered vaccinations intramuscularly (IM) for logistical ease and with a view to eventual roll out in resource limited settings.

The DNA and MVA-C were produced by UK HVC and based closely on those used previously (EV02 Eudract 2004-001802-28 and EV03 Eudract 2006-006141-13), with matched CN54/ZM96 subtype C-derived *gag pol nef* and *env* inserts. We anticipated Env-dominated CD4+ T-cell responses and modest Env-specific antibody responses after DNA and MVA, with the development of high titer binding and neutralizing antibody responses after boosting with adjuvanted CN54gp140 protein (29–31). Based on our previous studies, we expected that the immunogens would prove more potent B-cell immunogens than the ALVAC/AIDSVAX/ALUM used in RV144 and that the combined MVA/CN54p140/GLA might augment immunogenicity, offering the potential for a short regimen.

## Materials and Methods

### Study Design and Conduct

This was a Phase I randomized open-label trial conducted at two UK centers: Imperial College London and Surrey Clinical Research Centre. Participants were recruited through advertising, social media, and a dedicated website. The study documents were reviewed and approved by the NRES London—West London and GTAC Ethics Committee (13/LO/0115), and the UK Medicines and Healthcare products Regulatory Agency, and all participants gave fully informed written consent according to the Declaration of Helsinki before any study procedures were conducted. The trial was registered with the European Union Drug Regulating Authorities for Clinical Trials (EUDRACT TC 2012-003277-26) and Clinical Trials.gov (NCT01922284) and with the UK Clinical Trials Research Network (UKRN-14173). Laboratory personnel were blind to the allocation. Participants were block randomized centrally using a computer generated algorithm with a back-up manual procedure, and the randomization list was stratified by center and gender.

**Figure 1:**
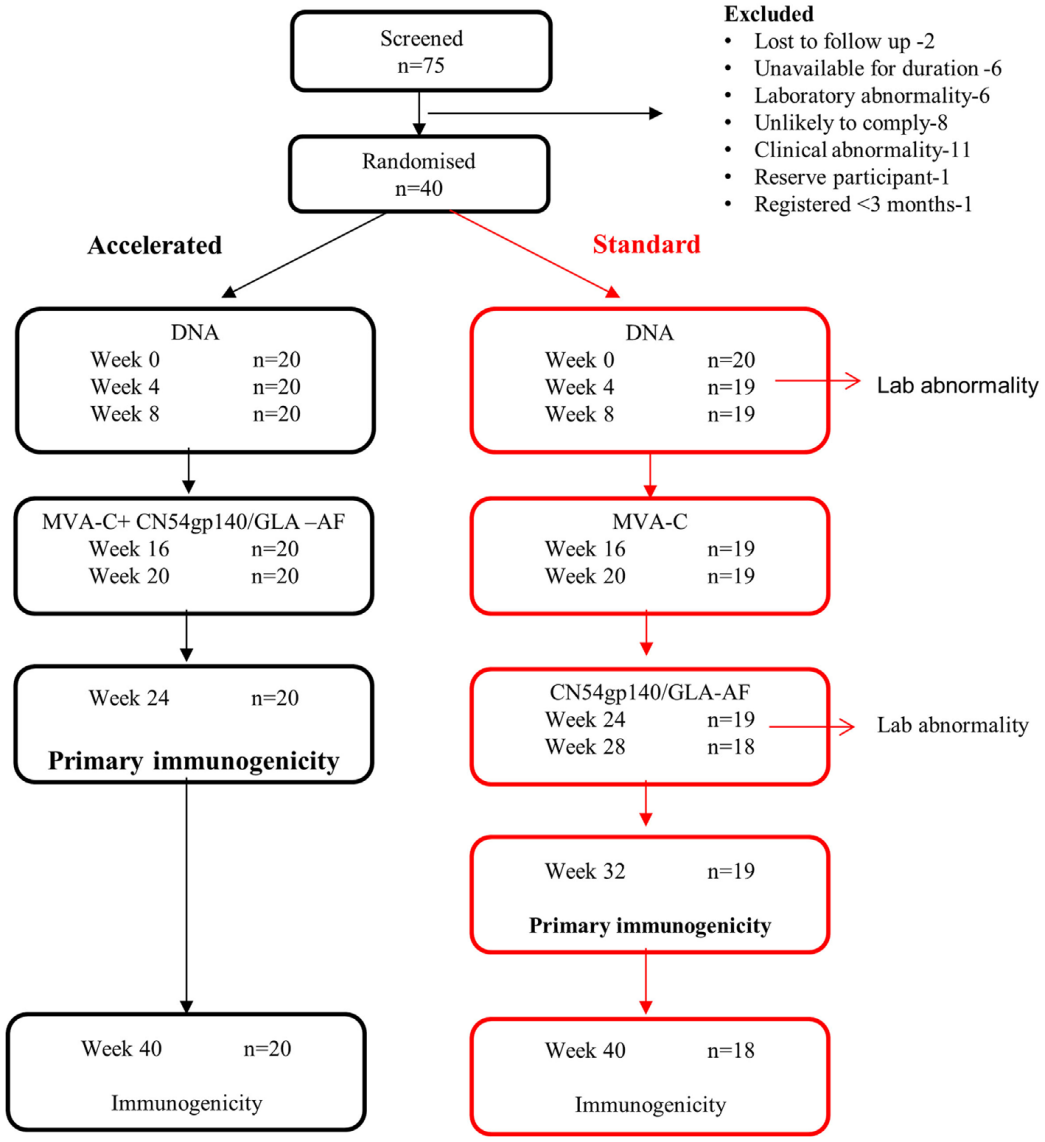
**Trial flow**.

The primary objective was to compare the safety and immunogenicity of two vaccination regimens, one of which was shortened by 8 weeks (Figure 1) in healthy HIV-uninfected male and female volunteers aged 18–45 years at low risk of HIV infection. The primary outcomes were (i) a severe (grade 3) or worse local or systemic clinical or laboratory adverse event or an event that led to a clinical decision to discontinue vaccinations and (ii) the magnitude of the CN54gp140-specific IgG antibody response in serum 4 weeks after the final immunization. Secondary outcomes of interest included IFNγ T-cell ELISpot, intracellular cytokine, neutralizing antibody, CN54gp140-specific serum IgA, mucosal IgG, and IgA antibody responses.

### Safety Evaluations

Local and systemic events recognized to be associated with licensed vaccines were solicited systematically at clinical centers prior to, 10 min and 1 h after each vaccination, and then 7 days later, and by diary card. Clinical and laboratory events were collected via an open question at each visit and through routine hematology and chemical pathology performed at screening, 1 week after each vaccination and at week 40 in both groups.

### Immunological Specimens

Blood was taken for immunological assessments at weeks 0, 4, 8, 16, 20, and 24 and 40 for all participants and at weeks 28 and 32 for those in the standard group. Mucosal samples were collected at weeks 0 and 24 for the accelerated and at weeks 0 and 32 for the standard group. Peripheral blood mononuclear cells (PBMCs) were isolated using density gradient separation, frozen in a mixture of fetal bovine serum (Sigma-Aldrich, St. Louis, MO, USA) and DMSO (9:1 ratio) using a Kryo 560-16 rate controlled freezer (Planer, Sunbury-On-Thames, UK). PBMCs were shipped and stored in vapor phase liquid nitrogen as previously (32). Genital tract secretions from women were collected using the Instead Softcup™ (Evofem Inc.) and urethral swabs (Hunt Biologics, UK) from male volunteers and rectal Floq™ swabs were taken when possible from males and females, primarily to assess the feasibility of the sampling method. Vaginal samples were collected, processed, and analyzed as described previously (33). Urethral swabs were collected from male participants in clinic by inserting the swab and allowing it to absorb mucosal secretions for 2 min. Rectal Floq™ swabs were inserted into the anus and rotated to collect secretions. Rectal and urethral swabs were either snap frozen on receipt or processed directly. Processing involved addition of 300 µl of extraction buffer [250 mM NaCl, 1× protease inhibitor cocktail set 1 (Calbiochem) in phosphate buffered saline (1× DPBS)] to the swabs, vortexing for 1 min and placing on ice to 15 min. Rectal and urethral swabs were then placed in the top chamber of a spin X tube, centrifuged at max speed (10,000 *g*) for 2 min and the eluates either analyzed immediately or aliquoted and frozen at −80°C until analysis.

### Vaccines and Schedule

The recombinant clade C HIV-1 envelope gp140 protein (CN54gp140) is a naturally cleavage resistant envelope clone of 97CN54 (34). The protein was manufactured to GMP specification (33) (Polymun Scientific Austria) generating a product which was >80% trimeric protein with a projected mass of 420 kD and a defined glycan (35). A total of 100 µg CN54gp140 was mixed with 5 μg GLA-AF (IDRI, Seattle, WA, USA) and administered in a volume of 0.4 ml as below. There were two DNA plasmids; one encoded (CN54) *env* and the other a (ZM96) *gag-pol-nef* fusion protein. Both open-reading frames were RNA and codon optimized (GeneArt AG, Regensburg, Germany). Both plasmids utilized a chimeric CMV enhancer/promoter with a human T-cell leukemia type 1 regulatory element to drive expression (36). The MVA-C (Mariano Esteban CSIC, Spain) expressed the CN54gp120 Env and Gag-Pol-Nef polyprotein from two back-to-back synthetic early/late transcriptional promoters (37, 38). All vaccinations were given IM into the deltoid muscles of the upper arms. 4 mg of each DNA plasmid was given to all participants at weeks 0, 4, and 8 in a volume of 1.0 ml (8.0 mg in total) with the same plasmid given into the same arm on each occasion (CN54 plasmid into right arm and ZM96 into left arm). In the “standard” group, 10^8^ TCID_50_ MVA-C was given at weeks 16 and 20 in a volume of 1.0 ml (into left arm) and then 100 µg CN54gp140 mixed with 5 µg GLA-AF at weeks 24 and 28 in a volume of 0.4 ml (into right arm). In the “accelerated” group, 10^8^ TCID_50_ MVA-C in 1.0 ml was given at the same time as 100 µg CN54gp140 mixed with 5 µg GLA-AF at weeks 16 and 20 in 0.4 ml IM as above (with MVA-C into left and CN54gp140/GLA-AF into the right arms as shown, see Table 1).

**Table 1:** **Schedule of doses, formulation, and routes of immunization**.

Group	Route of immunization; dose of vaccine		
	Weeks 0, 4, 8	Weeks 16, 20	Weeks 24, 28
1 (*n* = 20)	4 mg DNA (CN54) in 1 ml (right arm)	1 × 10^8^ TCID_50_ MVA-C in 0.5 ml (left arm) + [100 µg CN54gp140 + 5 µg glucopyranosyl lipid adjuvant—aqueous formulation (GLA-AF)] in 0.4 ml (right arm)	Nothing
	4 mg DNA (ZM96) in 1 ml (left arm)		
	Intramuscular (IM)	IM	

2 (*n* = 20)	4 mg DNA (CN54) in 1 ml (right arm)	1 × 10^8^ TCID_50_ MVA-C in 0.5 ml (left arm)	(100 µg CN54gp140 + 5 µg GLA-AF) in 0.4 ml (right arm)
	4 mg DNA (ZM96) in 1 ml (left arm)		
	IM	IM	IM

### Humoral Assays

#### CN54gp140-Specific Antibody ELISA

Serum and mucosal binding antibodies against recombinant CN54gp140 were measured using a standardized ELISA with minor modifications. 96-well ELISA plates were coated with 50 µl per well of capture antigen CN54gp140 (1 µg/ml) (Polymun, Austria). Human standards (IgG or IgA) were captured by coating wells with a combination of α-Human κ and α-Human λ (1:1 ratio) capture antibodies. After incubation at 37°C for 1 h, plates were washed with PBST then blocked for 1 h at 37°C with 200 µl/well of assay buffer (PBS + 1% BSA) then washed, as above (26). Standards were prepared by adding the required concentration of either human IgG or IgA. Serum samples were screened at 1:100 dilution, Softcup cervical mucosal samples at 1:10 dilution. Samples, standards, and controls (normal human sera) were added to triplicate wells. Detection antibodies were added following incubation and washing, either goat α-Human IgG-HRP or goat α-Human IgA-HRP detection antibodies. After incubation and washing, plates were developed by the addition of TMB substrate (KPL) followed by addition of 50 µl of Stop Solution (KPL). Absorbencies were read immediately at 450 nm using a VersaMax plate-reader (Molecular Devices). A response detected for both IgG and IgA was defined as OD A450 nm value >0.2; samples below this value were deemed negative or response not detected. Samples were further diluted following screening assays if positive with a series of dilutions in order to extrapolate a concentration expressed as microgram per milliliter of specific IgG or IgA using the ELISA software SoftMax Pro v 5.4. Serum samples that were positive by the above method were also tested in a conventional endpoint titer assay as previously described (32).

#### Neutralizing Antibody Responses

Neutralizing antibody responses against a panel of Tier 1 (MW965.26, MN.3, 00836-2.5, ZM197M-PB7) and Tier 2 (Ce1176_A3, Ce703010217_B6, HIV-2510-2) pseudo viruses were measured using TZM-bl cells in the lab of David Montefiori as described previously (27, 39). Briefly, pseudoviruses (TZM-bl assay) were incubated with serial dilutions of sera and added to their respective target cells. Luciferase expression was measured after 2 days (TZM-bl), and IC_50_s were determined as the serum concentration that reduced the background-subtracted relative light units by 50% compared to virus-only control wells.

### Cellular Assays

#### IFNγ ELISpot

Cellular immunogenicity was assessed by standardized IFNγ ELISpot assay using frozen PBMCs as previously described (32, 39). One day prior to assay setup, PBMCs were thawed in and rested overnight in RPMI medium containing 20% heat-inactivated fetal calf serum (HIFCS), glutamine, penicillin, and streptomycin (R20) (all supplied by Sigma, Poole, UK) at 37°C, 5% CO_2_. 96-well PVDF membrane (MSIPS4510 Millipore, UK) plates were coated with mouse anti-human IFNγ (10μg/ml; MabTech clone 1-D1K) in sterile PBS. On the day of assay setup, coated ELISpot plates were washed with sterile PBS and blocked with RPMI 10% HIFCS (R10) for at least 1 h. Synthetic peptides (15-mers overlapping by 11aa; HPLC purified >90%, JPT Germany) covering the HIV-1 gene inserts and CMV pp65 gene were dissolved and pooled in dimethyl sulfoxide (DMSO, Sigma), further diluted in PBS and R10 to achieve a final assay concentration of 1.5 µg/ml per peptide and 0.45% v/v DMSO. 100 µl volumes of HIV-1 peptide pools were added to ELISpot plate wells in quadruplicate. The CMV pp65 peptide pool and phytohemagglutinin (PHA, 10 µg/ml) were plated as positive controls in duplicate wells for each. For a negative control, quadruplicate wells containing a mock stimulus (0.45% v/v DMSO final concentration in R10) were used. Rested PBMCs were recovered and washed in R10 and viable cells counted using a Beckman Coulter Vi-Cell counter. A total of 200,000 viable PBMCs (in 50 µl) were added to all wells except for 1 well with R10 only (reagent control well). Plates were incubated at 37°C, 5% CO_2_ overnight (16–24 h). All subsequent steps were performed at room temperature. Plates were washed six times with PBS/0.05% v/v Tween 20 (Sigma) and the production of IFNγ by T-cells was assessed by addition of 1 μg/ml biotinylated mouse-anti-human IFNγ antibody (clone 7-B6-1, Mabtech, Sweden) for 2–4 h. Plates were washed as before and ABC peroxidase–avidin–biotin complex (PK6100, Vector labs, UK) was added for 1 h, followed by three washes with PBS/Tween and three washes with PBS. Spots were developed with addition of filtered AEC/H_2_O_2_ substrate solution (Sigma) for 4 min. The reaction was stopped by washing plates under running tap water, plate underdrains removed, and plates allowed to dry overnight in the dark before spots in each well were counted using an automated AID ELISpot reader (AutoImmun Diagnostika, Germany).

A positive response was defined by the following criteria: (1) average number of background-subtracted spots in a single pool >specified cutoff of 38 SFC/10^6^ PBMCs (40). The cutoffs were derived from assessing peptide pool responses in PBMCs from 178 HIV-1 seronegative individuals; (2) for each pool, if the number of replicates was 2 or ≥3, then the coefficient of variation (standard deviation/mean) between replicates had to be ≤50% or ≤70%, respectively; (3) mean count had to be >4 times mean background; (4) mean background had to be ≤55 SFC/10^6^ PBMCs. Samples with mean background >55 SFC/10^6^ PBMCs were considered failures, were repeated, and excluded from all analyses if failed a second time. The breadth of responses was described in terms of the number of individual peptide pools to which each individual responded.

### Flow Cytometry

Antigen-specific cytokine secretion was assessed using a validated seven-color polychromatic flow cytometry panel assessed at the IAVI human immunology lab in London. Previously frozen PBMCs were coincubated with peptide pools matched to the inserts at 1.5 µg/ml (as previously described), 1 µg/ml SEB (Sigma-Aldrich, St. Louis, MO, USA), or mock stimuli and Brefeldin A (Sigma-Aldrich, Poole Dorset, UK) for 6 h at 37°C. Cells were stained for viability with LIVE/DEAD Fixable Violet Dead Cell Stain Kit (Invitrogen, Eugene, OR, USA), fixed and then stained intracellularly using anti-CD4 PeCF594 (clone RPA-T4), anti-CD8 BV421 (RPA-T8), anti-CD3 APC-H7 (SK7), anti-IFNγ APC (B27), anti-IL2-PE (MQ1-17HI2), and anti-TNFα-FITC (Mab11) (all Becton Dickinson, San Jose, CA, USA). Cells were washed and acquired on the same day. At least 5,000 CD8 and CD4+ CD3+ viable, singlet lymphocyte events were acquired using BD Fortessa II instruments. Data were analyzed and presented using FlowJo (version 9.8 Treestar, Ashland, OR, USA). Samples were failed where fewer than 5,000 events in the predefined populations were acquired or where mock IFNγ responses were above 0.2% of either parental population. Flow cytometric analysis was performed at baseline, and 16 and 24 weeks for group 1, and additionally at 32 weeks for group 2.

### Peptide Array Mapping

The microarrays were processed according to the manufacturers instructions with minor modifications (www.jpt.com). Briefly, the slides were pre-incubated with T20 blocking buffer (Thermo Fisher) for 10 min. Plasma samples were then added at a dilution of 1:100 in T20 blocking buffer and incubated for 2 h at room temperature with gentle shaking before washing five times with 2.5 ml TBS-Tween (0.5% Tween). The secondary mouse anti-human-IgG Dylight649 (JPT) was then incubated at room temperature for 1 h at a dilution of 1:5,000 in T20 blocking buffer. After five washings with 2.5 ml TBS-Tween, and five washes with double distilled de-ionized water, the slides were left to dry under a laminar flow hood. Samples from all timepoints from one individual were processed simultaneously. Slides were scanned on a GenePix 4000A scanner and processed using GenepixPro 6.0 software at 650 and 532 nm to generate a Tiff image file. The array lay out was then added using the .gal file JPT_2758_V04.gal provided by JPT. Accuracy of the array alignment was controlled and individual features were adjusted or excluded manually when needed. After this QA/QC step, .gpr files were generated and processed further into .dat files using R-program and the R-script “MakeDat_V05r_stat.R” to generate one fluorescent intensity (FI) value for the peptide-specific IgG response from the triplicates. Individual IgG responses were mapped using the R-script “MapAlign_BG_V11.R” after subtraction of FI values from baseline plasma. The array included the immunogen sequence CN54gp140 and nine other sequences from acute phase primary HIV isolates of subtypes A, C, B, CRF01_AE, and CRF02_AG to maximize coverage of global HIV diversity (Ahmed *et al*., in preparation).

### Statistical Analyses

All clinical and routine laboratory data were included in the safety analyses. Data sets included (i) modified intention to treat; all participants who were randomized and received at least one vaccination and (ii) per protocol (PP), all participants who completed vaccinations with no major protocol deviations. The primary safety outcome was expressed as a proportion of participants with 95% confidence interval, and groups were compared using Fisher’s exact test. The primary immunological outcome was the magnitude of serum CN54gp140-specific IgG 4 weeks after the final vaccination and we assumed a 100% response rate in the standard (reference) arm. The sample size was calculated on the basis of the binding antibody responses distributions described in the RV144 trial. In this trial, the reciprocal GMT of binding antibodies to subtype E gp120 was ~1:15,000 (log10: 4.18) and for subtype B gp120 was ~1:30,000 (log10:4.5). On the basis of previous trials, we assumed that everyone would respond to the CN54gp140 and that the titer of subtype C-specific binding antibody responses would be in the same range and at least 1:15,000 in the standard group and that a four-fold increase in the magnitude of would be immunologically relevant. This translates to an absolute difference of 0.6 on the log10 scale. In the absence of raw data from the Rv144 trial, we have assumed a standard deviation of 0.58 on the log 10 scale in the distribution of the antibody responses (corresponding to a SD of ~33,400 in titer). Assuming this variation, 20 participants per group allowed for the detection of an absolute increase in titer of 0.60 with 90% power and 5% alpha. Comparison of the groups was made using the geometric mean (GM) ratios of the titer with their 95% CI and equality was assumed if these ratios included 1. Skewed data was log transformed for normality and then comparisons made using parametric tests. Secondary outcomes were compared by the response frequency per group, using chi-square tests if frequencies were adequate or the Fisher’s exact 2-tailed test for small numbers. Comparison of the magnitude of T-cell ELISpot responses was made using the non-parametric Wilcoxon two-sample test. No corrections were made for multiple testing. For the flow cytometric analysis, responses are described relative to each mock-stimulated control. Two-by-two contingency tables were generated to compare the peptide stimulated versus the mock control for each cytokine and T-cell subset. One-sided Fisher’s exact tests were then applied to each table to resolve whether the percentage of cytokines generated following peptide stimulation was greater than that compared following stimulation with mock antigen. Bonferroni corrections were applied to account for multiple testing. Heatmaps summarizing ICS analyses were generated using SPICE version 5.1 downloaded from http://exon.niaid.nih.gov (41).

## Results

### Participant Accrual, Study Population, and Compliance with Schedule

Of 75 healthy, low-risk, HIV-negative volunteers screened between 19th June 2013 and 10th January 2014, 40 were enrolled; the reasons for the 35 who were screened out are summarized in Figure 1. Twenty participants were enrolled at each center, and baseline characteristics are summarized in Table 2. The majority were white, half were female and the median age was 32 years (IQR 23–39). All randomized participants received the first immunization but two in the standard group did not complete the immunization schedule due to adverse events. In addition, two participants from the standard group missed the final visit at weeks 40 and 1 also missed the primary immunogenicity endpoint visit at week 32 (Figure 1).

**Table 2:** **Baseline characteristics and median follow-up by treatment group**.

	Accelerated	Standard	Total
	*n* = 20	*n* = 20	*n* = 40
Number	20	20	40
Age (SD)	31 (25–38)	32 (22–39)	32 (23–39)
Center			
Imperial College	10 (50%)	10 (50%)	20 (50%)
Surrey	10 (50%)	10 (50%)	20 (50%)
Gender			
Female	10 (50%)	9 (45%)	19 (47.5%)
Male	10 (50%)	11 (55%)	21 (52.5%)
Ethnicity			
Asian	2 (10%)	0 (0%)	2 (5%)
Mixed	0 (0%)	2 (10%)	2 (5%)
White	18 (90%)	18 (90%)	36 (90%)
Weight (kg)	73 (63–82)	72 (67–78)	72.8 (66–78)
Routine laboratory parameters			
Hemoglobin (g/dl)	14 (13–15)	14 (13–14)	14 (13–15)
White cell count (10^9^/l)	7 (5.3–8.2)	6.4 (5.8–8.0)	6.5 (5.6–8.2)
Neutrophils (10^9^/l)	4.1 (2.9–5.1)	3.6 (3.3–5.1)	3.8 (3.0–5.1)
Platelets (10^9^/l)	250 (236–288)	259 (225–289)	250 (219–288)
Lymphocytes (10^9^/l)	1.9 (1.6–2.3)	1.9 (1.6–2.3)	1.9 (1.5–2.3)
ALT (U/l)	20 (19–25)	19 (14–27)	21 (16–27)
AST (U/l)	21 (18–26)	23 (18–27)	23 (18–27)
Bilirubin (μmol/l)	10 (9–14)	11 (7–15)	10 (7–14)
Creatinine (μmol/l)	71 (58–78)	71 (59–84)	71 (60–80)
Glucose (mmol/l)	4.7 (4.4–5.0)	4.6 (4.5–4.9)	4.6 (4.4–4.9)
DNA/ANA antibodies			
Positive	4^a^ (20)	0	4
Negative	16 (80)	20	36
Follow-up (weeks, range)	43 (40–46)	45 (30–50)	44 (30–50)

*^a^Positive only at a dilution of 1:160 which was deemed eligible*.

### Primary Safety Endpoints

Four (20%; 95% CI 5.73–43.66) participants in the standard group and one (5%; 95% CI 0.13–24.87) participant in the accelerated group experienced a primary safety endpoint, *p* = 0.342 (two-tailed Fisher’s exact test). Two of these were ≥grade 3 laboratory adverse events that resulted in discontinuation of vaccinations. Both occurred in males aged 20 years in the standard group. The first occurred after the first DNA; the 7-day blood test revealed asymptomatic transaminitis (AST 375: Grade 3, ALT 109: Grade 2), which was confirmed 6 days later. Other blood tests, a viral screen, and ultrasound scan were normal. Apart from an episode of tonsillitis between screening and enrollment, treated with penicillin V, there were no other risk factors. The levels spontaneously returned to normal during the following week, but due to the temporal nature, a relationship could not be excluded and so vaccinations were discontinued. The second case occurred after administration of the second MVA-C. In between the immunization visit and the safety review, the participant reported an episode of vomiting after drinking around 12–14 units of alcohol, and strenuous exercise. The 7-day blood test revealed a transaminitis (AST 530: Grade 4, ALT 184: Grade 2). He was not able to return for 28 days at which time both were within the normal range. investigations including an ultrasound scan and blood tests for causes of viral and non-viral hepatitides were normal. Although it was felt that the more likely cause of the transaminitis was a combination of unusually high alcohol intake and strenuous exercise, there was a temporal relationship with vaccination and so vaccinations were discontinued. There were five others ≥grade 3 adverse events reported by three participants on diary cards during the 7 days after vaccination: severe malaise in a female accompanied by a headache 2 days after the first MVA-C/CN54gp140 GLA-AF, severe malaise in a male 5 days after the first CN54gp140 GLA-AF, and a severe/extreme lumpy swelling in a female 6 days after receipt of the first MVA-C and then again 3 and 6 days after the second MVA-C reported by the same participant.

There was one serious adverse event during the study; a female randomized to the accelerated group was referred to hospital by her GP with pain and suspected appendicitis 2 days after receiving her first combined MVA-C and CN54gp140 vaccination. She was treated with paracetamol for the pain (moderate grade according to the toxicity table) before being discharged after an overnight stay when the pain had resolved. The participant continued in the trial and received her last immunization without a repeat of this problem.

### Other Adverse Events

All of the remaining solicited local, systemic and other events were mild or moderate. There were 10 laboratory abnormalities other than those reported above, all of these were mild (5 raised ALT, 3 AST, 1 hyperglycaemica, and 1 bilirubin). There were differences in reporting between the centers (125 by Surrey; 50 by Imperial) and by gender (119 by females; 56 by males).

### Immunogenicity

All analyses presented below are derived from the PP data set and include 20 from the accelerated group and 17 from the standard group unless otherwise stated (1 participant from the standard group who received all vaccinations did not attend the primary immunogenicity visit and 2 did not receive all vaccinations).

### Primary Immunogenicity Endpoint

#### CN54gp140-Specific Binding Antibody

**Figure 2:**
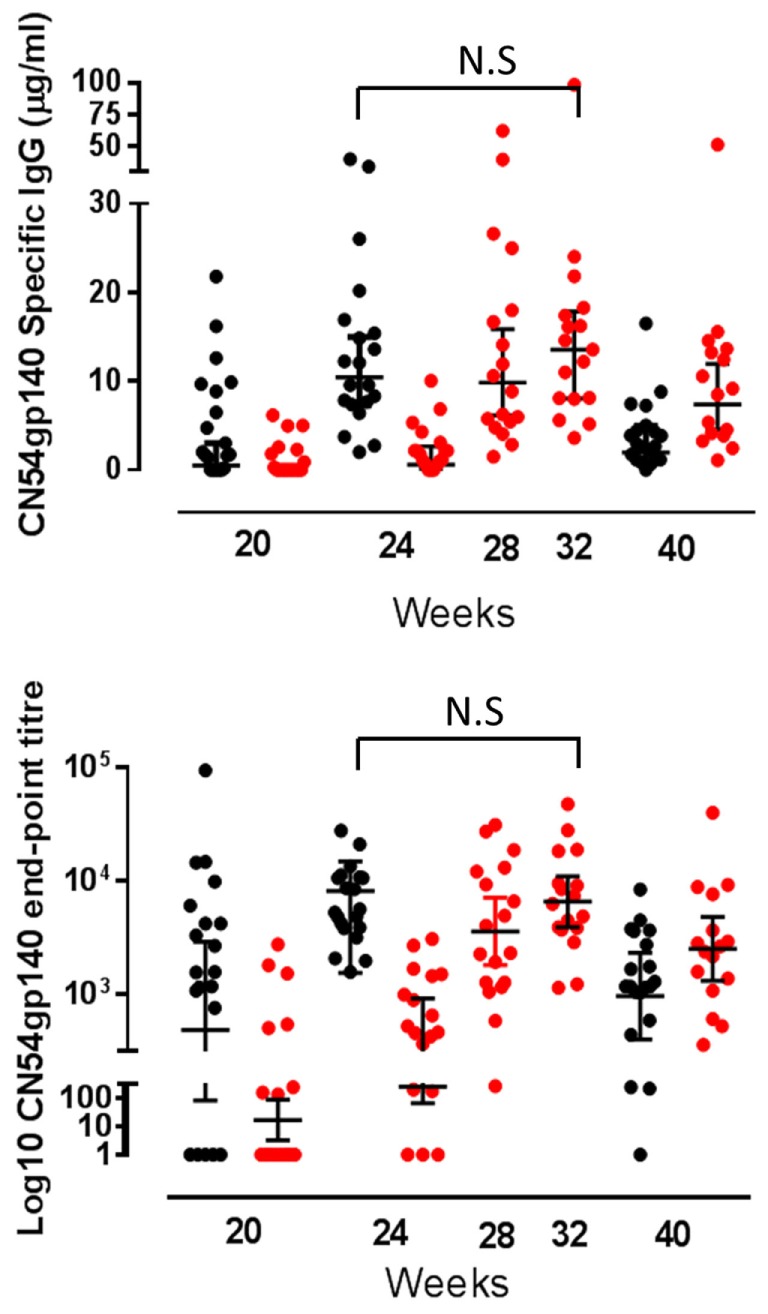
**Serum CN54gp140-specific binding antibody responses by group**. CN54gp140-specific serum IgG responses in accelerated (black closed circles) and standard groups (red closed circles). Solid lines represent geometric mean (GM) values with 95% CI. Comparisons made using the GM ratio of titers and concentration by group at the primary endpoint, and there were no significant differences between the groups (comparison of week 24 for accelerated group and week 32 for standard group).

At the primary endpoint 4 weeks after the final vaccination, all individuals who completed the schedule made CN54gp140-specific IgG. The GM titer and concentration of specific binding antibody in the accelerated group at the primary endpoint were 6,424 (95% CI 4,391–9,396) and 10.46 µg/ml (95% CI 7.3–15.0) and in the standard group 6,578 (95% CI 3,927–11,020) and 12.76 µg/ml (95% CI 8.7.0–18.75), respectively. There was no significant difference between the groups; as assessed by the ratio of the GM titer (0.98, 95 CI 0.53–1.79, *p* = 0.93) or concentration (1.46, 95% CI 0.49–4.34, *p* = 0.49). The response was first detected in both groups 4 weeks after the first MVA-C (week 20) when 15/20 (75%) in the accelerated and 8/18 (44.4%) in the standard group were positive—with a greater response in the accelerated group, *p* = 0.02. In both groups, the response increased significantly after each subsequent vaccination (Figure 2). In the accelerated group, the GMT at week 20 was 517, and this increased to 6,424 at week 24. In the standard group, the GM titer was 17 at week 20, increasing to 246 at week 24, 3,596 at week 28, and 6,578 at week 32. In both groups, the response fell away again by week 40; to GMT 1,302 and 2,536 in the accelerated and standard groups, respectively, a difference which was significant (*p* = 0.02) (Figure 2). There was no CN54gp140-specific serum IgA detected at any time point.

#### Mucosal Antibody Responses

**Figure 3:**
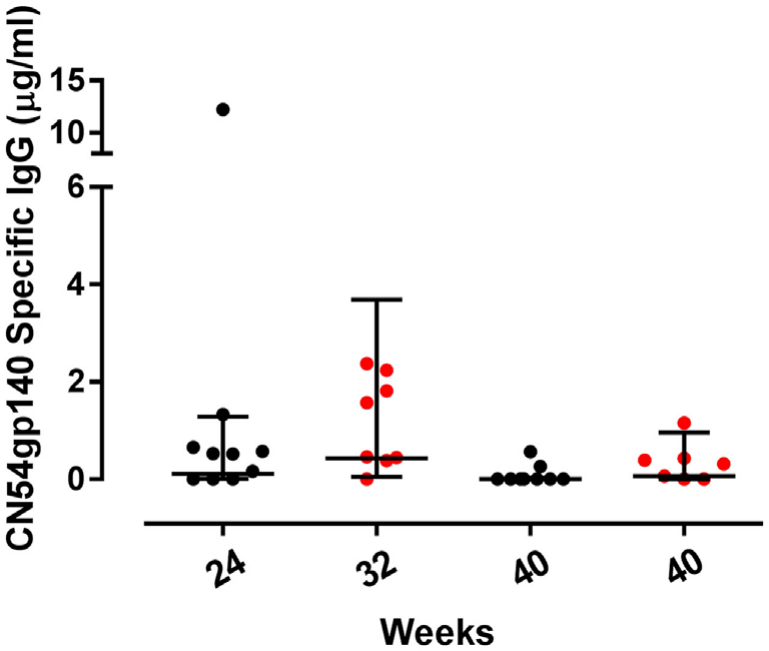
**Mucosal binding CN54gp140-specific binding antibody responses by group**. Concentrations of CN54gp140-specific cervicovaginal IgG responses at the primary endpoint at week 24 for accelerated (black circles) and week 32 for standard groups (red circles) and at week 40 for both. Solid lines represent geometric mean (GM) values with 95% CI. Comparisons were made using non-paired *t*-tests using GM values, and there were no significant differences between the groups.

Of the mucosal sites sampled, CN54gp140-specific IgG was only detected in cervicovaginal secretions, with no specific responses detected in either urethral or rectal samples (Figure 3). The only samples included in the analyses of mucosal secretions were collected from women using Instead cups. There was no CN54gp140-specific IgG detected in samples at baseline, and at the primary endpoint there was no difference in the frequency of responders between the groups; 70% (7/10) women had detectable CN54gp140-specific IgG in the accelerated group as compared to 88% (7/8) in the standard group (*p* = 0.59, Fisher’s exact test). The GM concentration of cervicovaginal CN54gp140-specific IgG was 0.11 µg/ml (95% CI 0.01–1.28) in the accelerated group compared with 0.43 µg/ml (95% CI 0.05–3.7) in the standard group 4 weeks after the final vaccination. There was no CN54gp140-specific IgA detected in cervicovaginal samples.

#### Neutralizing Antibody Responses

**Figure 4:**
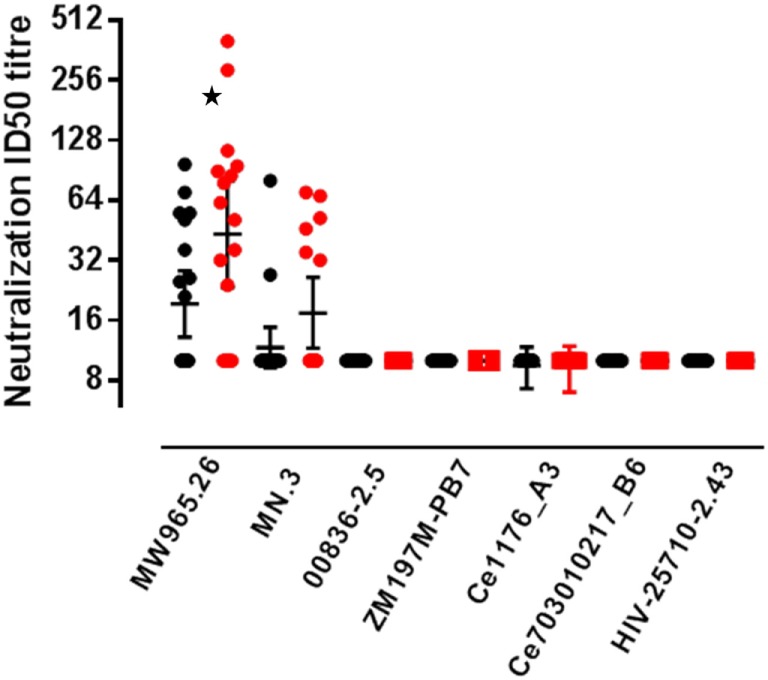
**Serum neutralizing antibody responses by group**. Neutralizing antibodies measured in serum from accelerated and standard groups at the primary endpoint, 4 weeks after the final vaccination: accelerated group, closed black circles (measured at week 24) and standard group, closed red circles (measured at week 32). Virus strains; MW965.26 (Clade C, Tier 1A), MN.3 (Clade B, Tier 1A), 00836-2.5 (Clade C, Tier 1B), ZM197M-PB7 (Clade C, Tier 1B), Ce1176_A3 (Clade C, Tier 2), Ce703010217_B6 (Clade C, Tier 2), and HIV-25710-2.43 (Clade C, Tier 2). Solid lines represent geometric mean titer with 95% CI. The frequency of responders in each group was compared using the Fishers exact test, *p = 0.04.

Neutralizing antibody responses were detected against two Tier 1A Env-pseudoviruses (Figure 4). At the primary endpoint 9/20 (45%) participants in the accelerated group showed neutralization of subtype C MW965.26 virus (closest match to CN54gp140) compared to 14/17 (82%) in the standard group, a difference which was statistically significant (*p* = 0.04, Fisher’s exact test) with higher median titer neutralization in the responders from the standard relative to the accelerated group (median titer of 51 and 78, respectively). There was also a trend toward a higher frequency response to MN.3 (Tier 1 subtype B) in the standard group (10 versus 35% positive, *p* = 0.107). There was no neutralization detected against two Tier 1B Clade C viruses (00836-2.5, ZM197M-PB7) or against 2 Tier 2 clade C viruses (Ce1176_A3, Ce703010217_B6, HIV-25710-2.43).

#### Peptide Array Mapping of the Env-specific Antibody Response

**Figure 5:**
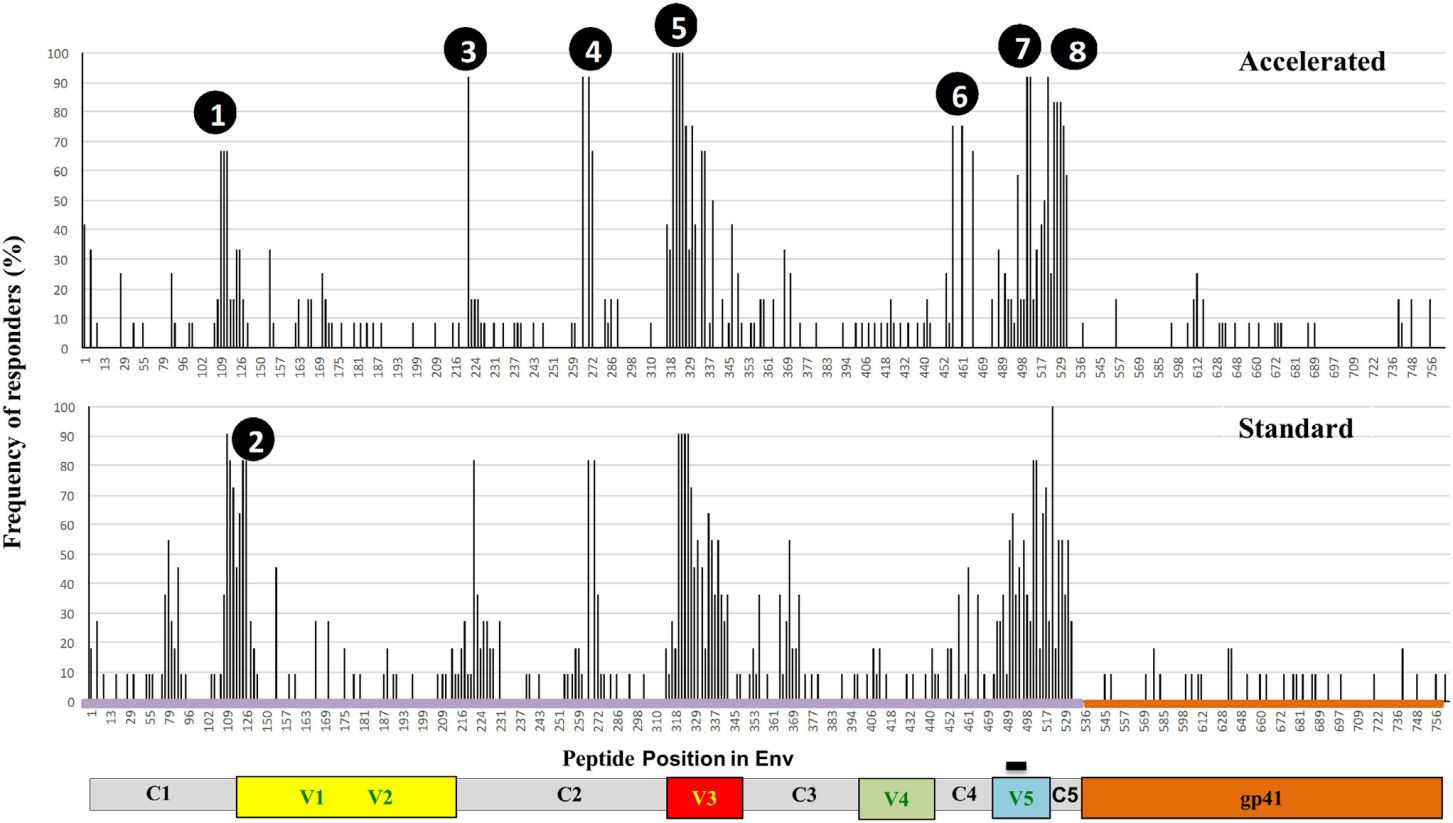
**Specificity of systemic binding antibody responses to Env by group**. Frequency of recognition of linear overlapping peptides spanning the HIV envelope in plasma samples from the accelerated (*n* = 12, upper panel) and the standard groups (*n* = 11, lower panel). The *y* axis shows the proportion of individuals recognizing the specified peptides and the *x* axis denotes the distribution of linear peptides along the envelope with relevant regions of interest highlighted.

Vaccine-induced Env-specific IgG responses to linear 15-mer peptides (Table 3) were mapped using a custom designed peptide micro array approach in subjects of accelerated group (*n* = 12) and standard group (*n* = 11) 4 weeks after the final vaccination. The immunodominant regions (IDRs) targeted and magnitude of region-specific responses were largely similar between the two groups (Figure 5). IDRs were exlusively located within gp120 with little recognition of gp41. Basic characteristics of IDR-specific IgG responses including the representative peptide sequence targeted and mean fluorescence intensity within each group are summarized in Figure 5. Within gp120, four consecutive peptides covering the tip of the V3 region (indicated as peak 5, aa300 to 320 (HxB2 reference strain) were targeted by >90% of vaccinees with a high mean fluorescence intensity (MFI) (referring to the highest measured response, if multiple peptides covered for this region) of above 50,000 units in both groups. Other IDRs were located in the C1 region (peak 1; HxB2 aa position 104–124 and peak 2; HxB_aa117–136) with maximum MFIs of 43,000 and 25,000 in standard and accelerated groups, respectively; in the C2 region (peak 3; HxB_aa200–215 and peak 4; HxB_aa245–264) with MFIs between 25,000 and 35,000; and in the C4 (peak 6 HxB2_aa429–457) and C5 regions (peak 7, HxB2_aa473–490 and peak 8, HxB2_aa491–504) were targeted with MFIs between 20,000 and 38,000. All these IDRs were recognized by 70% of vaccines in at least one group. There was no significant differences between the groups in terms of the number of epitopes recognized, the magnitude of individual responses or the sum of fluorescence intensity values for all peptide variants recognized that were included in the array (*p* = 0.21).

**Table 3:** **Antigenic peaks of recognition in Env**.

Peak number	HXB2	Env region	Representative sequence	MFI standard	MFI accelerated
1	104	C1a	MHEDIISLWDQSLKP	34650	19507
1	107	C1a	DVISLWDQSLKPCVK	43478	25183
1	109	C1a	ISLWDQSLKPCVKLT	41274	21634
2	119	C1b/V1	SVKLTPLSVTLNSTD	11993	x
2	121	C1b/V1	KLTPLCVTLNCTNAK	29195	x
3	200	C2	AITQACPKVTFDPIP	30593	25445
4	245	C2	VQCTHGIKPVVSTQL	33780	36173
4	249	C2	HGIKPVVSTQLLLNG	15699	20643
5	300	V3	GNNTRKSIRIGPGQT	44475	40678
5	301	V3	NNTRKSIHIGPGQAF	60570	59711
5	304	V3	RKSINIGPGRAFYAT	59915	59186
5	305	V3	TSIRIGPGQTFYATG	59589	56526
6	429	C4	EVGKAMYAPPIKGQI	x	15762
6	433	C4	AMYAPPIKGQIKCLS	x	19922
7	473	C5	GDMRNNWRSELYKYK	34577	22160
7	475	C5	MKDNWRSELYKYKVV	37522	22782
8	491	C5	IKPLGVAPTTTKRRV	35026	35758

*Summary of HxB location, Envelope region, representative peptide sequences, and mean fluorescence intensity (MFI) of the most frequently recognized antigenic regions in Env displayed in Figure 5 and the MFI of each peak from standard (*n* = 11) and accelerated (*n* = 12) groups*.

#### T-Cell ELISpot Responses

**Figure 6:**
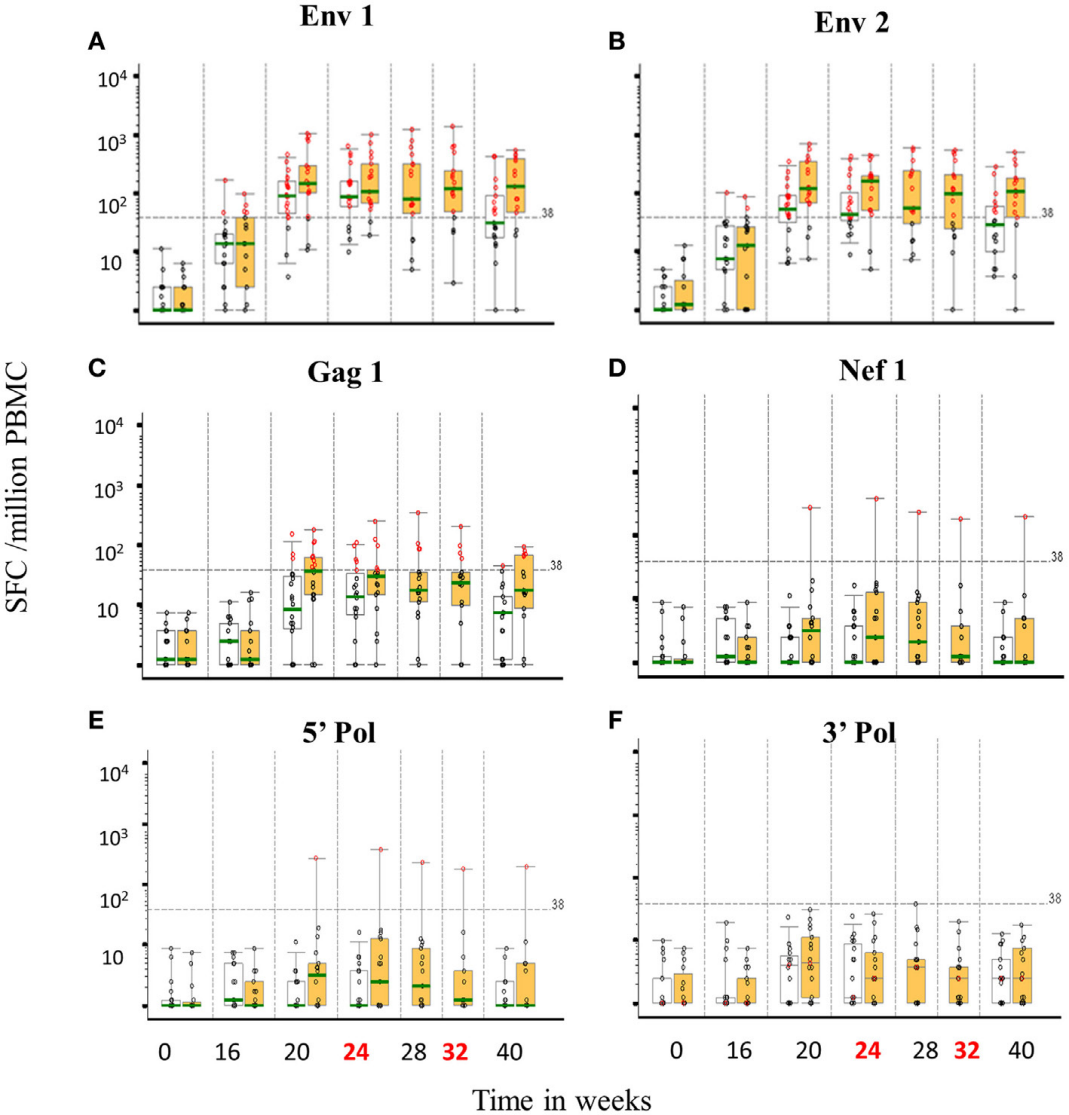
**T-cell ELISpot responses by group over time to vaccine encoded peptide pools**. Distribution of IFNγ ELISpot responses (background subtracted; spot forming units per million PBMCs) prior to and following vaccine candidate administration for six HIV-1 peptide pools; CN54 1/2, Env 1 **(A)** and 2 **(B)**, ZM65 Gag **(C)**, Nef **(D)**, 5′ Pol **(E)**, and 3′ Pol **(F)**. Boxes represent the interquartile ranges, whiskers extend to the 5th and 95th percentiles and the green bar is the median. Red circles represent positive responses, black circles are negative responses. Accelerated group: open boxes, *n* = 20, Standard group: orange boxes, *n* = 18. Dashed line is the ELISpot assay positive response value (38 SFU/million PBMCs).

There were no IFNγ ELISpot responses at baseline and 8 weeks after the third DNA vaccination (week 16) responses were seen in a minority of individuals to Env peptide pools (20.5% overall). At the primary endpoint, there was no difference in either the frequency or magnitude of ELISpot responses between groups; 85 and 82.4% responded to any pool in the accelerated and standard groups, respectively, at the primary endpoint (*p* = 1.00) with overall GM values of 111 and 147 SFC/10^6^ PBMCs (*p* = 0.44) Figure 6. All responding individuals recognized one or both Env peptide pools (75.0 and 76.5% recognizing Env pool 1 and 65.0 and 64.7% recognizing Env pool 2 for the accelerated and standard groups, respectively, *p* values >0.99) and the magnitude of these responses was similar between groups [medians 70–189 SFC/10^6^ PBMCs for the two Env pools (*p* = 0.09–0.32)]. Gag peptide-specific responses were relatively modest and detected in only 25% of the accelerated and 23.5% of the standard group (*p* = 1.00) with similar magnitude (median 58 and 86 SFC/10^6^ PBMCs, *p* = 0.28). There were no responses to the 5′ Pol pool in either group and none of the accelerated group and 17.6% of the standard group responded to the 3′ Pol pool (*p* = 0.09, median 43 SFC/10^6^ PBMCs). None of the accelerated group and 5.9% of standard group responded to Nef peptides (*p* = 0.46, 1 response of 179 of SFC/10^6^ PBMCs in the standard group). At this time point, the mean number of peptide pools recognized (out of a total possible of 6) for each subject was similar between groups; 1.65 and 1.88 for the accelerated and standard groups respectively (median of 2 for both groups, *p* = 0.63). In terms of magnitude, responses to Env pools peaked 4 weeks after the second MVA-C/CN54gp140 (median 111 SFC/10^6^ PBMCs) in the accelerated group and 4 weeks after the first MVA-C in the standard group (median 213 SFC/10^6^ PBMCs). While the great majority of responses in both groups recognized Env or Env plus Gag peptides, recognition of ENV in combination with Nef or 3′Pol or of Gag peptides was only seen in the standard group—accounting for approximately 17% of all responses (data not shown). In the accelerated group, responses to Env and Gag peptides dropped between weeks 24 and 40 (*p* = 0.04, 0.02) whereas in the standard group there was no significant change (*p* = 0.4, 0.65). At week 40, the responses to Env and Gag peptide pools was of greater magnitude in the standard than the accelerated group (*p* = 0.03, 0.02, and 0.091, respectively) and more frequent to Env peptides, with 71% responding to both Env peptide pools in the former compared to 37 and 47% in the latter (*p* = 0.09 for responses to Env 1 pool) and this was also true of responses to Gag peptide pools with 30% responding in the standard and 5% in the accelerated group.

#### Intracellular Cytokine Responses

**Figure 7:**
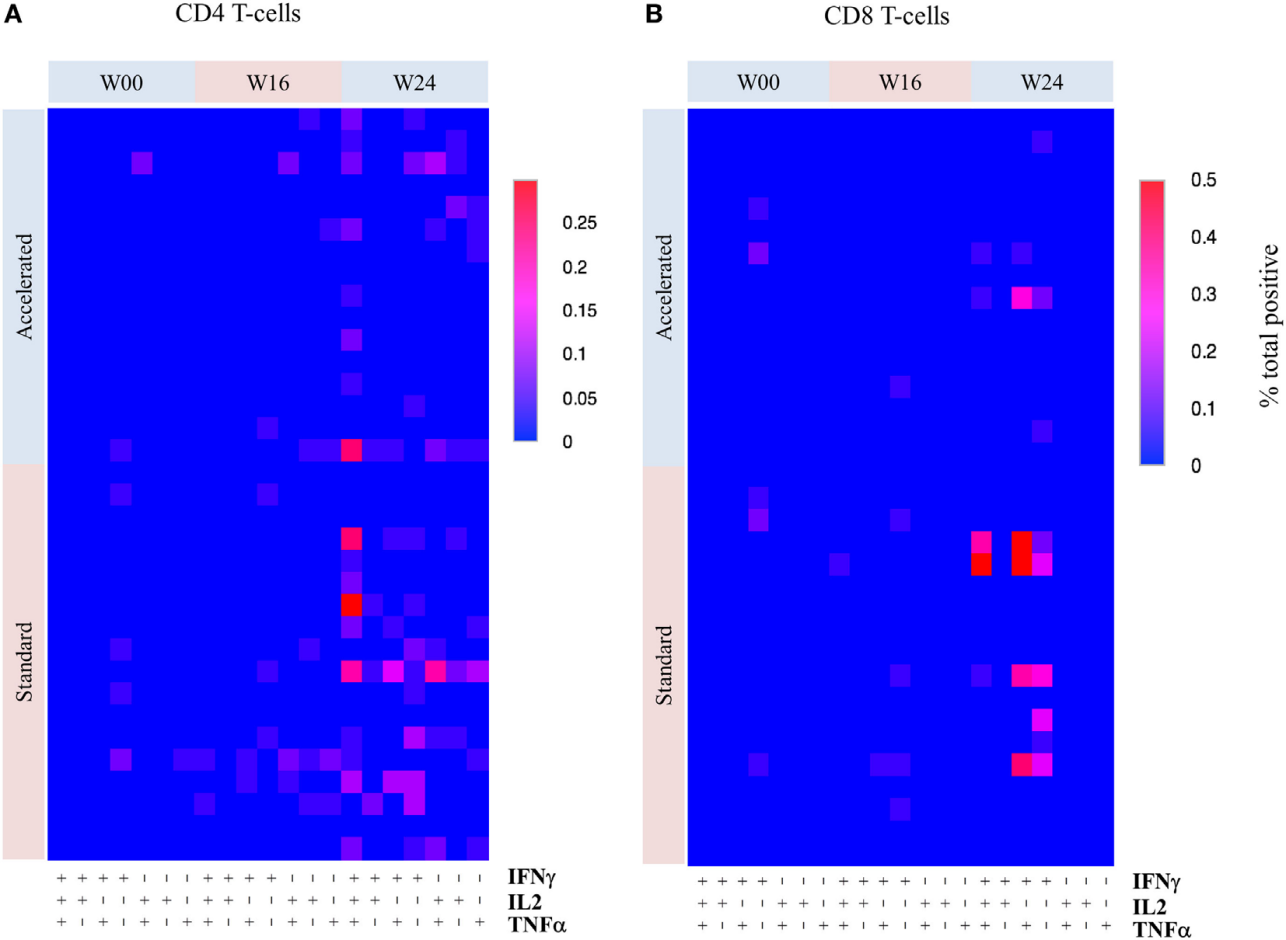
**Heatmaps showing the frequency of different cytokine responses by T-cell subset over time by group**. Overall frequency of different combinations of three cytokines (IFNγ, IL2, and TNFα) produced by CD4 **(A)** and CD8 **(B)** T-cells responses to combinations of P5/6 Env peptide pools by week. The highest frequency (% of total) is shown in red and the lowest in blue (scale depends on maximal response). Due to the low frequency of responses, no formal statistical comparisons were made between the groups.

Overall, intracellular cytokine responses were modest and results are therefore descriptive. The majority of responses were polyfunctional and focused toward Env rather than Gag peptide pools. Response rates to *any* antigen at *any* post-baseline visit, for both CD4 and CD8 populations, were higher in the standard than the accelerated group, 50 versus 30% respectively for CD4+, and 33.3 versus 10% for CD8+ lymphocytes, but these differences were not statistically significant (data not shown). CD4 and CD8 responses to all antigens were predominantly polyfunctional, with IFNγ being the dominant cytokine (Figure 7). The standard group tended toward higher CD8 responses (IFNγ+TNFα+) while both groups had comparable CD4 responses (the majority being IL2+IFNγ+). In the standard group, responses seemed to peak at week 24 and wane at week 32 in CD4 lymphocytes but were more persistent in some CD8 lymphocytes (data not shown).

## Discussion

We have compared two vaccination regimens using identical DNA-C, MVA-C, and GLA adjuvanted CN54gp140 with the aim of assessing the safety and immunogenicity of a shortened regimen in which the MVA and adjuvanted protein were combined. We expected strong Env-specific CD4+ T-cells after DNA and MVA and Env-specific binding antibodies in everyone after adjuvanted gp140 and the study was powered to detect a four-fold difference in the magnitude of this response between groups. As expected, 100% individuals made strong CN54gp140-specific antibody irrespective of regimen, but combining the vaccines had no detectable impact on the magnitude or specificity of the antibody response as assessed by the recognition of linear peptides. This pattern was characterized by strong recognition of V3 with notably little recognition within gp41—in spite of its presence in the DNA and gp140 vaccines. The immunogens also induced recognition of linear epitopes within C2 and C4, but their significance, if any, is unknown. Despite these binding antibody responses, neutralizing antibody responses were disappointing and only seen to Tier 1A pseudoviruses, similar to our previous experiences using this adjuvanted protein (27, 28). Interestingly, however, the frequency and titer of the responses was inferior when vaccines were combined to two Tier 1A pseudoviruses. There was no difference in the overall frequency or specificity of T-cell ELISpot responses which were seen in >80% individuals irrespective of group and tended to be CD4+ and specific for Env peptide pools. While the great majority of IFNγ ELISpot responses were seen to Env peptide pools in both groups, recognition of Gag, Nef and Pol peptides was less frequent in the accelerated group suggesting that combining the vaccines might have led to further polarization of immune responses. Cytokine responses were relatively modest, but also less frequent or less polyfunctional in both CD4+ and CD8+ compartments when the MVA and CN54gp140 were combined. There was no significant impact of combining the vaccines upon tolerability. Although two participants experienced events that resulted in discontinuation of immunizations, these followed different immunogens (DNA-C, MVA-C) and there were alternative explanations for the asymptomatic elevation in transaminases in each case. The great majority of adverse events were mild in line with our previous experience using the same adjuvanted protein in different settings (27, 28).

Even though we controlled for variables such as site and gender, the study has limitations; overall statistical power was compromised by the fact that we did not have 40 in the final analyses as planned. Nevertheless, there was clear evidence that combining MVA and gp140 led to attenuation of certain T-cell and B-cell immune responses. Both regimens were shorter overall than used in previous studies using similar homologous (22–24) or heterologous DNA and MVA (26, 42) and it remains possible that the time between DNA prime and MVA boost (8 weeks) might have been too short to allow for the optimal maturation of immune responses. The use of a common and semi-quantitative assay for the measurement of CN54gp140-specific IgG antibody allows for direct comparison across our different trials. The median CN54gp140- specific binding antibody response seen here (12.8 µg/ml) exceeds that seen in the Mucovac 2 trial (UK HVC_001) after three doses of CN54gp140/GLA-AF IM in the absence of DNA priming (4.2 µg/ml) (27), but is lower than seen in the TaMoVac 01 trial (UK HVC_00 2) after boosting twice with GLA-AF adjuvanted CN54gp140 30–71 weeks after priming with DNA and MVA (17.8 µg/ml) (28). This supports the value of DNA/MVA priming and suggests that the long gap between prime and boost could be important. Sallusto and colleagues propose a minimum gap of 12 weeks and note that if boosting is too frequent, responding cells might be preferentially driven to terminal differentiation resulting in attenuation of immunity (43).

Our decision to use immunogens expressing matched subtype C inserts was driven by our commitment to a vaccine for use in Sub-Saharan Africa as well a belief that this approach would ellicit high titer binding antibodies and so favor functional/neutralizing antibodies. In addition to the logistical advantages offered by fewer vaccinations, we were interested in combining the pox and protein in light of the results of the RV144 trial which included combined canarypox and alum adjuvanted gp120 and was the first ever trial of an HIV vaccine to show (modest) efficacy. In UK HVC_003 overall, immunogenicity was somewhat disappointing and it remains possible that the combined MVA and adjuvanted gp140 protein may have overwhelmed the pool of antigen-specific/innate immune cells, as offered as one explanation of the attenuation in responses sometimes seen when certain pediatric vaccines are combined (44, 45) and which might be particularly associated with MVA at it has been shown to be highly immunogenic and to preferentially deplete antigen presenting cells (46–48). Immunogenicity may have been further compromised in the accelerated group as a result antigenic competition (even though the vaccines were administered into opposite arms) as has been suggested to occur in response to response to certain combinations of conjugated vaccines (49) and observed more recently in an HIV vaccine trial in South Africa (25). The choice of adjuvant was partly practical as we had access to GLA-AF through the UK HVC and had already used it with the same gp140 protein (27, 28). In our hands, the GLA-AF (MPLA) adjuvant has previously been shown to be potent for antibodies at equivalent (3 × 100 µg) and lower (3 × 20 µg) doses (27) (in the absence of DNA/MVA), and we had every reason to suspect that priming would further enhance these responses. We expected the immunogens to be at least as immunogenic as those used in RV144 in both groups and, based on our previous experiences with DNA and MVA, predicted that they would be more potent for T-cells.

In light of these findings, we remain cautious about the accelerated regimen, and feel that the combined MVA-C and adjuvanted GLA-AF warrants further evaluation in a regimen with a longer gap between priming and boost. We believe this trial provides further support for exploring the clinical efficacy of a priming regimen including DNA, with at gap of least 12 weeks prior to boosting. The precise contribution of adjuvanted protein (if any) is yet to be defined. While Churchyard and colleagues reported no clear benefit of DNA priming on Env-specific antibody responses, T-cell responses appeared augmented by the priming although overall immunogenicity in the trial was lower than seen previously using the same vaccines in a different population (25). In light of our previous experience with a variety of immunogens, we remain committed to the inclusion of DNA as we believe balanced immune responses highly desirable. We await data from a direct comparison of the specificity of antibody responses from trials using different heterologous prime boost regimens (including RV144 and Tamovac 01) with this one to inform our selection of immunogens to take forward. Non-neutralizing IgG antibody responses targeting V2 have been shown to correlate with a reduced risk of HIV using a variety of different approaches, although we did not see much response to linear peptides within this region (9, 50, 51) suggesting perhaps that they are associated with the subtype E gp120 protein. We did however see strong recognition of the V3 loop in both groups and this has been described as characteristic of the responses seen during natural infection (27) and also in other vaccine trials including RV144-when the response correlated with reduced risk of HIV acquisition in a subset of individuals (51). In conclusion, the vaccines were potent irrespective of regimen, but immunogenicity was lower than anticipated, and we cannot exclude the possibility that this was due to the relatively short regimens. Our data suggest that combining MVA and CN54gp140/GLA-AF in this relatively short regimen had no significant impact on safety and also no impact on the magnitude of CN54gp140-specific systemic antibody responses and the strategy may have attenuated immunogenicity as reported previously (25).

## Author Contributions

SM, JW, RS, SJ, JG, DL, RW, and ME oversaw and/or designed the study/immunogens. KQ, AG, SM, AM, SJ, AC, RT, and DL were involved in the day to day running and conduct of the study. AC, PM, PH, JK, CG, YN, MA, and DM were involved in laboratory analyses, interpretation of results, and drafting figures. LD, SJ, and GB conducted the statistical analyses. SJ drafted the manuscript with editorial support and comment from PM, PH, SM, AC, RS, CG, JW, DM, RW, ME, LD, and GB.

## Conflict of Interest Statement

The authors declare that the research was conducted in the absence of any commercial or financial relationships that could be construed as a potential conflict of interest.
